# Genome Sequence of Acinetobacter baumannii Strain SHOU-Ab01, Isolated from Chinese Giant Salamander (Andrias davidianus) Liver

**DOI:** 10.1128/mra.00503-22

**Published:** 2022-07-12

**Authors:** Lu Yuan, Xiaoyan Zhang, Yufei Lyu, Yan Guo, Jie Chen, Dongshu Wang, Liping Wang, Hengliang Wang, Xiankai Liu

**Affiliations:** a College of Food Sciences and Technology, Shanghai Ocean University, Shanghai, China; b State Key Laboratory of Pathogen and Biosecurity, Beijing Institute of Biotechnology, Beijing, China; University of Maryland School of Medicine

## Abstract

This report describes the complete genome sequence of Acinetobacter baumannii strain SHOU-Ab01, which was isolated from the liver of a Chinese giant salamander (Andrias davidianus). SHOU-Ab01 belonged to sequence type 40 (ST40), and its genome contained a circular chromosome (size, 3,891,862 bp) and two circular plasmids (sizes, 8,571 bp and 5,870 bp).

## ANNOUNCEMENT

Acinetobacter baumannii can cause serious infections in hospitalized patients and aquaculture animals (1). In this study, A. baumannii was isolated from the liver of a Chinese giant salamander (Andrias davidianus), and whole-genome sequencing and analysis were performed.

Each part of the Chinese giant salamander was aseptically dissected, and the samples were homogenized using a Stomacher 3500 beat-type homogenizer (Seward, England). The samples were diluted 10^−6^ by 10-fold gradient dilution with 0.9% NaCl, spread on LB agar, and cultured at 30°C for 24 h, and then a single colony was streaked and cultured (2). Strain SHOU-Ab01 was isolated from the liver, and its basic morphology was identified using a Gram staining kit (Solarbio, China). Genomic DNA from a SHOU-Ab01 culture in LB liquid medium at 37°C for 8 h was extracted using the Wizard genomic DNA purification kit (Promega, USA), following the instruction manual (3, 4). The DNA concentration and quality were assessed using a NanoDrop 2000 spectrophotometer (Thermo Fisher Scientific, USA) (5).

Whole-genome sequencing was performed using a combination of Pacific Biosciences (PacBio) Sequel II sequencing and next-generation sequencing via the Illumina NovaSeq 6000 platform. PacBio data were assembled using HGAP4 and Canu (v1.6). Pilon 1.22 (v1.24) software was used to correct the PacBio assembly data using the next-generation sequencing data (6). NovaSeq 6000 sequencing data with 150-bp paired-end reads (total number of reads, 7,500,000), and FastQC (v0.11.5) was applied to analyze read quality. The PacBio system was used for single-molecule real-time (SMRT) sequencing, with an average read length of 14,493 bp, an *N*_50_ value of 17,680 bp, and a total of 128,610 reads. PacBio data itself have been quality controlled, and no additional quality control analysis is required. The genome sequence was annotated by using the National Center for Biotechnology Information (NCBI) Prokaryotic Genome Annotation Pipeline (PGAP) (v5.3) (7), which revealed 3,746 gene sequences, including 3,558 protein-coding genes, 93 pseudogenes, 18 rRNA genes (5S, 16S, and 23S), 73 tRNA genes, and 4 noncoding RNA (ncRNA) genes. Software was used with the default settings and parameters unless otherwise specified.

**FIG 1:**
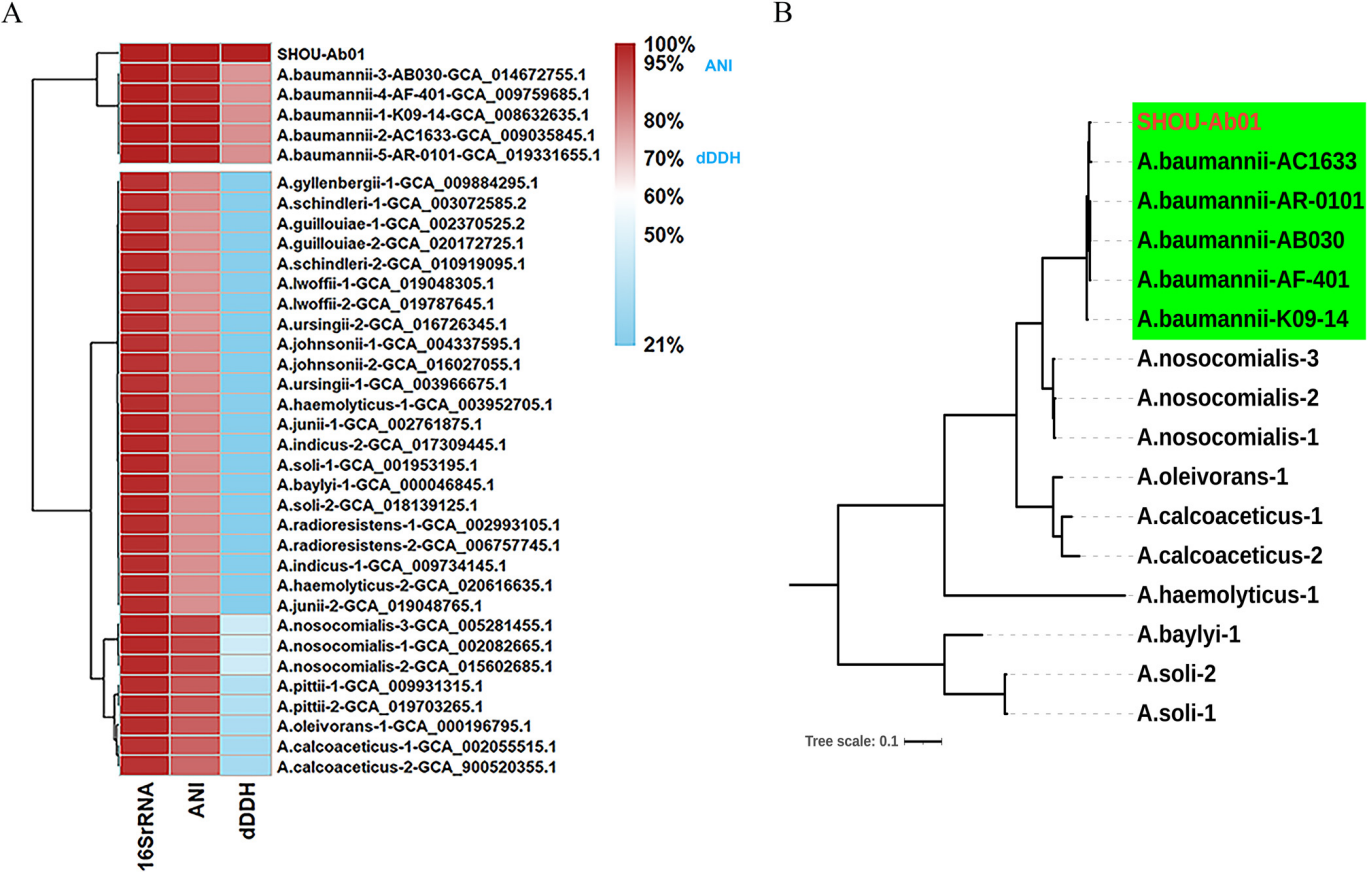
Phylogenetic relationships of strain SHOU-Ab01. (A) After 16S rRNA gene sequence analysis and calculation of ANI and dDDH values, the 16S rRNA gene sequence similarity (≥98.7%), ANI (≥95%), and dDDH (≥70%) values indicated that strain SHOU-Ab01 belongs to the species A. baumannii. (B) The whole-genome single-nucleotide polymorphism (SNP) cluster analysis showed that strain SHOU-Ab01 belongs to the species A. baumannii.

In Fig. 1, the 16S rRNA sequence similarity, average nucleotide identity (ANI), and digital DNA-DNA hybridization (dDDH) values were calculated using BLAST (v2.7.1+) local service (8), FastANI (v1.3) local service (9), and the Genome-to-Genome Distance Calculator (GGDC) (v3.0) (https://ggdc.dsmz.de/ggdc.php), respectively (10). These 16S rRNA sequence similarity (≥98.7%), ANI (≥95%), and dDDH (≥70%) values (10) indicate that strain SHOU-Ab01 belongs to the species A. baumannii. The Pasteur multilocus sequence typing (MLST) sequence type (ST) was analyzed using seven housekeeping genes (*cpn60*, *fusA*, *gltA*, *pyrG*, *recA*, *rplB*, and *rpoB*), which indicated that strain SHOU-Ab01 was ST40 (11).

Genomic analysis of A. baumannii SHOU-Ab01 may provide insights into the genetic basis of A. baumannii and antibacterial prevention for giant salamander breeding.

### Data availability.

The genomic sequences are available in NCBI GenBank under BioProject accession number PRJNA781087. The raw reads for PacBio and next-generation sequencing have been deposited in the SRA database under accession numbers SRR18933346 and SRR18919489, respectively. The strain attributes and accession numbers are provided in Table 1.

**TABLE 1:** Assembly statistics and genome features of A. baumannii strain SHOU-Ab01

Parameter	Data from:		Data for:		
	Illumina NovaSeq 6000	PacBio Sequel II	Chromosome	Plasmid pAb01-1	Plasmid pAb01-2
Assembly statistics					
Total no. of reads	7,500,000	128,610			
Avg read length (bp)	150	14,493			
Read *N*_50_ (bp)		17,680			
SRA accession no.	SRR18919489	SRR18933346			
BioSample accession no.	SAMN23235066	SAMN23235066			
Genome features					
Total length (bp)			3,891,862	8,571	5,870
Coverage (×)			272.54	559.88	1485.85
GC content (%)			39.01	35.47	35.13
Total no. of genes			3,725	12	9
GenBank accession no.			CP087594	CP087595	CP087596
